# Analysis of the Veterinary Risk Assessment and Management Plan questionnaire responses for dairy herds enrolled in the Northern Ireland Johne's disease control programme

**DOI:** 10.1002/vro2.71

**Published:** 2023-10-09

**Authors:** Kayleigh Meek, Sam Strain, Niamh E. O'Connell, Irene R. Grant

**Affiliations:** ^1^ Institute for Global Food Security School of Biological Sciences Queen's University Belfast Belfast Northern Ireland UK; ^2^ Animal Health and Welfare Northern Ireland Dungannon Tyrone Northern Ireland UK

## Abstract

**Background:**

Animal Health and Welfare Northern Ireland has been enrolling dairy herds across Northern Ireland (NI) in a voluntary Johne's disease (JD) control programme since October 2020. A Veterinary Risk Assessment and Management Plan (VRAMP) questionnaire was completed for each herd enrolled and recommendations for improved farm management practices were provided to farmers. Herd JD testing was recommended but was not mandatory.

**Methods:**

This study analysed VRAMP responses for 1569 dairy herds that had enrolled in the JD control programme up to October 2022. Univariate and multivariate regression models were applied to the data as appropriate.

**Results:**

Overall, 21.4% of the dairy herds had completed herd JD screening, with 13.7% of herds reporting a confirmed case of JD. A further 31.5% of herds reported suspected case(s) of JD. Eighty‐nine percent of farms had introduced animals from outside the herd. Herds that utilise a mixed calving pen and hospital pen, and herds that do not separate JD‐positive or sick animals within the calving pen, were significantly (*p* > 0.001) more likely to be a high‐probability JD herd. Accidental mixing of neighbouring herds significantly (*p* = 0.01) increased the risk of a suspected or confirmed case of JD. Herds that utilise rented land (70%) were significantly (*p* > 0.001) more likely to be at a high risk for JD.

**Conclusions:**

The VRAMP analysis identified areas of JD control that should be focused on in NI dairy herds, such as calving pen management and hygiene. The results highlight the importance of common JD recommendations in the management of on‐farm disease risk.

## INTRODUCTION

*Mycobacterium avium* subspecies *paratuberculosis* (MAP) is the causative agent of Johne's disease (JD), which occurs endemically in dairy cattle. Johne's disease in cattle presents as chronic granulomatous enteritis, causing clinical signs such as decreased milk production, wasting, lethargy and diarrhoea, eventually leading to mortality.^1^, ^2^ Due to the development of clinical signs, dairy producers must contend with substantial economic losses.

The Northern Ireland (NI) dairy sector has primarily extensive, pasture‐based farms, with animals housed over winter, grazed on pasture from spring to autumn; most farms utilise spring calving systems, with some farmers choosing all‐year calving. In 2021, approximately 318,000 dairy cows were farmed in NI on 2562 farms,^3^ which was approximately 17% of dairy cattle in the UK.^4^ In 2012, Animal Health and Welfare Northern Ireland (AHWNI) was launched as a province‐wide body that works alongside farmers’ organisations and veterinarians to promote improved cattle health and welfare to livestock producers and processors. The AHWNI body implemented a voluntary Johne's disease control programme (JDCP) across the province in 2020; herdowners enrolled themselves onto the programme via their veterinarian or directly through AHWNI. The JDCP focuses on three main goals: bio‐exclusion, biocontainment and market reassurance. Bio‐exclusion aims to identify herds that are currently free from JD and provide professional support to ensure that the farms stay JD negative. Biocontainment aims to provide support to herds that test positive for JD and assist with controlling and reducing the herd prevalence of disease.^5^

Once a herd is enrolled, authorised veterinary practitioners (AVPs) who are specifically trained by AHWNI for the JDCP visit carry out what is referred to as a Veterinary Risk Assessment and Management Plan (VRAMP). The VRAMP is designed to assess each herd's individual JD risk and consists of general herd management and JD risk‐related questions. At the end of the visit, the AVP provides up to three personalised recommendations for disease control. Alongside the VRAMP, it is recommended that each farm undergo full herd JD screening, with a view to making this compulsory in the future.

The objectives of this study were to ascertain the prevalence of risk factors for JD in NI dairy herds and to identify which high‐risk on‐farm practices were more prevalent on NI farms that previously had a confirmed or suspected case of JD.

## METHODS

### Data collection and study sample

Herds were included in the study if they were voluntarily enrolled in the AHWNI JDCP and had completed a VRAMP between August 2020 and October 2022. A total of 1569 dairy herds were eligible for the study sample. Fifty herds had completed two VRAMP questionnaires by October 2022. The most recent VRAMP for these herds was excluded from the analysis, ensuring that all analyses were carried out on initial VRAMP data only (so that any changes farmers may have made in light of veterinary practitioner recommendations after their first VRAMP would not impact the analysis).

The VRAMP is a risk assessment tool that is used to assess and quantify a range of infection risks associated with JD.^5^ It was designed using previous reports of JDCPs^6^ and adapted to Irish and Northern Irish dairy industry management practices.^7^, ^8^ It was extensively trialled to ensure that the questions were fit for the purposes of identifying infection risks and providing a mechanism for an informed discussion between a herd owner and their veterinary practitioner.^7^ The VRAMP is focused on environmental, biosecurity and biocontainment risks; it does not claim to encompass all possible risk factors related to JD. For example, dam associative risk factors^9^ were not covered in the VRAMP. Only veterinarians who had completed bespoke training by AHWNI on JD and the NI JDCP were permitted to carry out the VRAMPs.

The questionnaire information was collected electronically via a bespoke online web form using Wufoo (www.wufoo.com, Momentive) an online form building platform. All the data collected were stored securely within the Wufoo platform. Each herd's data are only available to the herdowner, their nominated veterinarian and to AHWNI. The data were downloaded by AHWNI from Wufoo as a CSV file and anonymised before being analysed. There were no incomplete VRAMPs, so none had to be excluded from the analysis.

### Veterinary Risk Assessment and Management Plan question details

The VRAMP questionnaire consisted of an initial section containing six questions centred on the herd's previous JD testing status and a second section containing 34 questions relating to JD risk factors and biosecurity measures (see Supporting Information S1). For the first section, questions were either closed answer questions with two response options offered (yes [Y] or no [N]) or multiple choice questions where additional information was required.

The biosecurity and risk section of the VRAMP was further split into two sections. The first section comprised a mix of multiple choice, closed answer (Y/N) and open‐ended questions similar to section one, whereas the second section comprised of a series of matrix questions that asked the AVP to rate risk factors on a scoring scale (1, 4, 7 or 10), with ‘1’ being scored if the farmer does not undertake these practices, or if the way in which the practices are undertaken has minimal risk in relation to JD, and ‘10’ being scored if the farmer undertakes the practice in such a way that could substantially increase JD transmission risk in the herd. The guidance provided to veterinarians carrying out the VRAMP on how to score risk factors is given in the Supporting Information S2.

### Descriptive and statistical analysis

The automatically collated data from Wufoo were exported into Microsoft Excel. Risk values of ‘1’, ‘4’, ‘7’, ‘10’ were renamed in Excel as ‘1’, ‘2’, ‘3’ and ‘4’, respectively; ‘Y’ and ‘N’ were renamed in Excel as ‘2’ and ‘1’, respectively. For frequency and descriptive analysis, risk values of ‘1’ and ‘4’ were categorised as low to medium risk and ‘7’ and ‘10’ as high to very high risk. All questions were given a short name identifier to enable identification of each variable in R. Answers to open‐ended questions requiring numeric responses were assigned to groups; for example, answers to the question ‘How many animals have you brought into the herd in the past year?’ were assigned to the following groups: ‘0’, ‘1–99’ and ‘100 or more’. Once the data were organised, descriptive analysis and frequency statistics were carried out in Microsoft Excel.

For statistical analysis, herd data were split into two categories: high probability of JD present and low probability of JD present. The ‘high probability of JD present’ category included herds that answered ‘yes’ to either ‘Have you had any suspected cases of clinical Johne's disease, for example, cows with chronic diarrhoea/chronic wasting?’ or ‘Have you ever had a confirmed case of Johne's disease in your herd?’. These were herds that had previously had a confirmed faecal culture or PCR‐positive case of JD or had reported animals with suspicious clinical signs. ELISA results were not available for consideration alongside the VRAMP analysis, so herds that had ELISA‐positive animals might not be included within this category; however, we cannot assume these did not exist. These herds had a higher probability of having JD present at the point of completing the VRAMP.

The ‘low probability of JD present’ category included herds that answered ‘no’ to either ‘Have you had any suspected cases of clinical Johne's disease, for example, cows with chronic diarrhoea/chronic wasting?’ or ‘Have you ever had a confirmed case of Johne's disease in your herd? (Faeces test positive)’. These herds had never had a positive faecal culture or PCR test result for JD; furthermore, they had not reported any animals with suspicious clinical signs. These herds had a lower probability of having JD at the point of completing the VRAMP. However, limited testing and potential gaps in farmer knowledge meant that answering no to these questions does not necessarily mean that the herds were infection free at the point of completing the VRAMP.

Statistical analyses of the risk‐related data were performed using R (version 4.1.0)^10^ and RStudio (version 1.4.1717).^11^ Additional software programs/packages readxl,^12^ car,^13^ epitools,^14^ corrplot^15^ and tidyr^12^ were used in R. Univariate Pearson's chi square analysis of nominal and ordinal data was completed against data that were categorised as ‘high probability of JD present’ herds. The univariate Mann–Whitney *U*‐test was used for ratio data. Odds ratios and confidence intervals were calculated for independent variables with *p*‐values less than 0.15 in the univariate analysis, comparing a control value (risk factor of 1 × ‘low probability’ herd) to all other values. Independent variables with *p*‐values less than 0.15 in the univariate analysis were also included in a logistical regression model with a manual backwards elimination with a forwards step applied, as previously described.^16^ This meant that significant variables (*p* < 0.05) after the logistical regression model were retained in the final model. Spearman's correlation was then used to check for co‐linearity between independent variables which were retained with rho values greater than 0.3 reported. A correlation plot between all numerical variables excluding descriptive data (such as QUB ID) was produced. Data analysis of follow‐up questions, which were only available if the answer to a previous question was ‘Yes’, were analysed within the ‘Yes’ group using Pearson's chi square and odds ratios as above.

## RESULTS

### General herd data and risk factor‐related descriptive analysis

**FIGURE 1:**
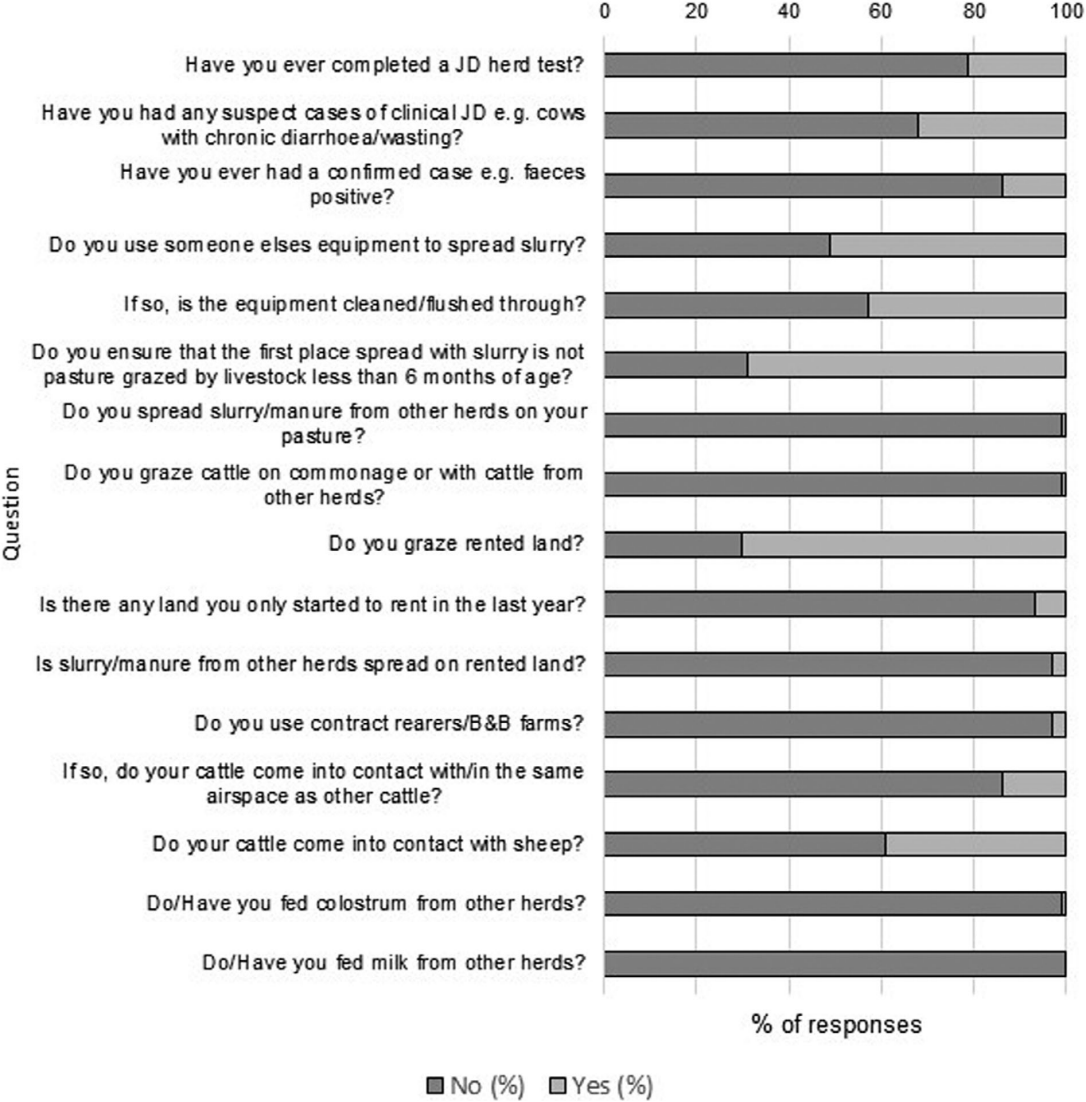
Analysis of frequency data for all closed questions with the options of ‘yes’ or ‘no’ from the Veterinary Risk Assessment and Management Plan results for 1569 dairy farms. JD, Johne's disease.

Figure 1 outlines the frequency data for all closed questions with the options of ‘yes’ or ‘no’ and shows the percentage of herds that had previously undertaken a JD test. Table 1 shows the distribution of different types of herd testing. The highest percentage of herds undertook individual milk sampling (67.6% of herds that answered yes to having previously undertaken testing, 14.5% of all herds enrolled in the study) and the testing with the lowest uptake was faecal culture (4.5% of herds that had previously undertaken testing, 1.0% overall).

**TABLE 1:** Distribution of different types of herd Johne's disease testing (%), based upon herds that answered ‘yes’ to having previously been tested and based on total herd numbers (*n* = 1569).

If you have had a herd test, what test(s) have you used?	Percent of herds which have had a herd test	Percent of herds overall (*n* = 1569)
Individual milk—ELISA	67.6	14.5
Individual blood—ELISA	24.7	5.3
Bulk milk—ELISA	20.5	4.4
Faecal culture	4.2	0.9
Faecal PCR	4.5	1.0
No test used	N/A	78.6

Of the 1569 herds in the study, 29.8% had previously had a suspected clinical case of JD, of which 68.5% had a suspected case within the past 3 years and 21.6% had a suspected case during the most recent year of VRAMP collection. Of herds with a suspected case, 38.3% had observed more than four suspected cases in the herd.

### Common risk factors

Table 2 outlines data concerning biosecurity risks related to the purchase of animals, with 89.4% of herds reporting at least one animal into the herd. Farmers had purchased from a median of three different herds within the past 5 years. Fifteen percent of herds experienced accidental mixing with neighbouring herds two or more times within the past year. Responses showed that cattle came into contact with sheep, either on the home farm or on contract farms, in 39% of herds. Grazing cattle on common grazing land or with cattle from other herds occurred in 1% of herds.

**TABLE 2:** Distribution of results from biosecurity risk‐related Veterinary Risk Assessment and Management Plan questions outlining the percentage, median and range of results from all herds (*n* = 1569).

Question	Percent of herds with a result >0	Median from all herds results	Minimum, maximum
Estimate how many animals you have brought into the herd in last 5 years?	89.4	10	0, 2500
Estimate how many herds you have purchased from in last 5 years?	88.5	3	0, 1025
Estimate how many purchased animals were kept for breeding?	84.5	10	0, 700

**FIGURE 2:**
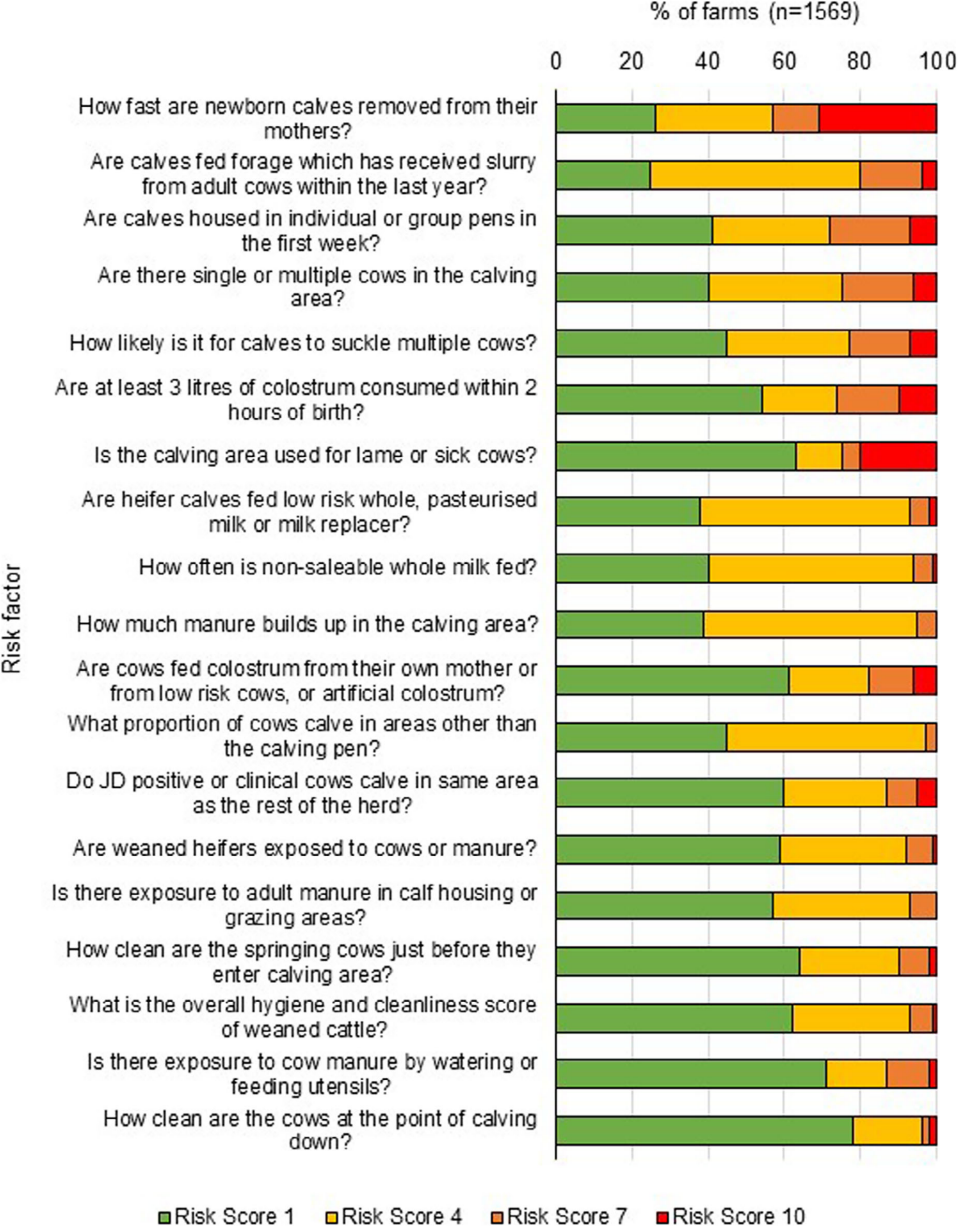
Distribution of risk scores (1, 4, 7 or 10) assigned by authorised veterinary practitioners for Johne's disease (JD) risk factor questions in the Veterinary Risk Assessment and Management Plan questionnaires for 1569 herds enrolled in the Northern Ireland JD control programme from August 2020 to October 2022. Risk factors are ranked from highest to lowest average risk score. The scoring scale is colour coded to represent risk score: red for a score of 10 for highest risk practice, green for a score of 1, representing good practice and yellow and orange for risk scores of 4 and 7, respectively, for increasingly risky practices.

Risk factors related to the use of slurry and slurry spreading equipment showed that over half (51%) of herds in the study used slurry spreading equipment, which was hired from either another farm or a contractor. Of these farmers, 43% responded that the equipment used was not cleaned or flushed through between farms. When using hired slurry equipment, 69% of farmers responded that they did not ensure that the first field that slurry was spread onto was not pasture grazed by livestock less than 6 months of age (Figure 1). A large majority (70%) of herds grazed rented land, while only 7% of herds had land that they only started to rent in the year prior to VRAMP data collection (Figure 1). Almost one‐third (28%) of herds had a high to very high‐risk scores of 7 or 10 for feeding silage, other conserved forages or grass to calves that had received slurry or farm manure from adult cows within the 12 months prior to data collection. However, high‐risk scores for exposure to adult manure were minimal across all risk factors (Figure 2).

Herds had a higher risk score in relation to what milk was fed to heifer calves, with 25% of herds feeding milk considered to have a higher JD risk, such as whole milk from individual cows without selecting for JD test status or whole milk from multiple cows from a bulk tank. A smaller percentage of farms (18%) were scored as higher risk for JD because they were likely to feed non‐saleable whole milk to calves in the herd occasionally or routinely, with 6% of herds feeding non‐saleable milk every week. In over 90% of herds, over 50% of calves were likely to consume approximately 3 L of colostrum within 2 h of birth and were fed low‐risk colostrum, for example, from their own mother, a low‐risk cow or artificial colostrum (Figure 2).

Twenty‐one percent of herds were reported to have no segregation of high‐probability JD clinical, test‐positive or suspect animals from low‐probability animals at calving (Figure 2), with a higher percentage of herds (25%) using the calving area to house lame or sick cows once a month or more. A similar percentage (23%) of herds scored high to very high risk scores, of 7 or 10, due to housing more than one cow in the calving pen more than 25% of the time. In relation to removal time of calves from the dam, 30% of herds scored a very high risk (10), where less than 10% of newborn calves were removed from the dam within 30 min of birth. Seven percent of herds scored a high or very high‐risk score for cows calving in non‐designated areas, for example, in areas where there was a high risk of exposure to adult faeces such as cubicle houses (more than 5% of calvings within the year prior to completing the VRAMP).

### Biosecurity, bio‐exclusion and biocontainment risk analysis

The results of the univariate analysis comparing risk factors with herds that were categorised as ‘high probability of JD present’ for all variables with a *p*‐value of less than 0.15 are reported in Table 3. Odds ratios are presented as risk factor level against the lowest risk factor level, for example, ‘1’.

**TABLE 3:** Retained associations (*p* < 0.15) between biosecurity risk factors and herds with a high probability of Johne's disease (JD) presence from the Veterinary Risk Assessment and Management Plan analysis (1569 herds).

Risk factor	*n*	Odds ratio	*p*‐Value	Confidence interval
**Are there single or multiple cows in the calving area?**				
1—If there is never more than one cow in the calving pen/area.	701			
4—If <25% of the time, there is more than one cow in the calving pen/area.	500	1.58	<0.001	1.23–2.03
7—If 25%–50% of the time, there is more than one cow in the calving pen/area.	250	1.53	<0.001	1.12–2.08
10—If more than 50% of the time there is more than one cow in the calving pen/area.	118	2.50	<0.001	1.67–3.73
**Are cows fed colostrum from their own mother or from low risk cows, or artificial colostrum?**				
1—If all calves receive fresh clean colostrum only from their own test negative mother, or are fed colostrum from known low risk cows or artificial colostrum (this is only recommended in emergency situations), and if all calves born to a test positive mother are removed from the breeding programme permanently.	929			
4—If all calves receive clean colostrum from only their own mother (no selection but no exposure of calves to cows other than their own mother) or are fed from a single low risk ‘donor’ cow (selected because test negative and older [≥8 years of age] and a record kept linking recipient calf to donor cow).	512	1.08	0.48	0.86–1.37
7—If colostrum from another cow(s) (pooled or frozen), with no selection based on JD status, is fed to 1%–10% of calves.	111	1.78	0.005	1.18–2.65
10—If colostrum from another cow(s) (pooled or frozen) with no selection based on JD status is fed to more than 10% of calves.	17	0.99	0.96	0.30–2.72
**Are calves housed in individual or group pens in the first week?**				
1—Calves are housed in single pens and away from the main cubicle/housing/calving area.	1120			
4—Calves are housed in single pens in the main cow cubicle or housing/calving area.	255	1.43	0.01	1.08–1.90
7—Calves are housed in groups ≤9.	165	1.42	0.04	1.00–1.99
10—Calves are housed in groups ≥10.	29	1.48	0.31	0.67–3.15
**What is the overall hygiene and cleanliness score of weaned cattle?**				
1—If heifers have no manure visible on hindlegs, forelegs or flanks.	705			
4—If manure is present on hind or forelegs but not above hock/carpal joints.	810	1.18	0.14	0.95–1.47
7—If manure is present on hind or forelegs above hock/carpal joints and/or is present on the flanks.	53	2.12	0.02	1.13–3.96
**How clean are the springing cows just before they enter the calving area?**				
1—If no cows have manure visible on hindlegs, forelegs or flanks.	610			
4—If manure is present on hind or forelegs but not above hock/carpal joints.	878	1.16	0.20	0.93–1.44
7—If manure is present on hind or forelegs above hock/carpal joints and is present on the udder or flanks of a few cows (<10%).	65	1.97	0.009	1.16–3.31
10—If manure is present on hind or forelegs above hock/carpal joints and is present on the udder or flanks of a significant proportion of cows (>10%).	7	1.02	0.98	0.13–5.01
**How much manure builds up in the calving area?**				
1—Pen is cleaned out between each calving.	397			
4—No visible manure, pen has not been cleaned between every calving, but new bedding is added so that the bedding is dry.	858	1.53	<0.001	1.17–2.02
7—Visible manure covering some of the floor (less than 50%).	251	2.06	<0.001	1.46–2.91
10—Visible manure covering most of the floor (more than 50%).	63	2.96	<0.001	1.71–5.14
**Is the calving area used for lame or sick cows?**				
1—If the area is NEVER used by non‐calving cows.	626			
4—If the area is used rarely (e.g., once a quarter).	542	1.21	<0.001	0.93–1.56
7—If the area is used occasionally (e.g., once a month).	302	1.91	<0.001	1.42–2.56
10—If the area is used routinely (e.g., it is the usual place where sick/lame/treated animals are held).	99	2.91	<0.001	1.88–4.50
**Do JD‐positive or clinical cows calve in the same area as the rest of the herd?**				
1—No JD clinical, test positive, suspect or high‐risk cows have contact with calving pens used by other cows. Such high‐risk cows are calved in a separate location.	948			
4—Rarely a test positive, suspect or high‐risk cow calves in the general calving area but never any animal showing clinical signs.	178	3.72	<0.001	2.43–4.69
7—Frequently test positive, suspect or high‐risk cow calves in the general calving area but never any animal showing clinical signs.	110	3.08	<0.001	1.94–4.91
10—There is no segregation at calving between high risk JD clinical, test positive, suspect or high‐risk and low‐risk cows.	333	1.79	<0.001	1.36–2.36
**What proportion of cows calve in areas other than the calving pen?**				
1—If no calves are born anywhere other than in the designated calving area/pen/on clean grass in the last year.	599			
4—If 0%–5% of calvings in the last year occurred outside the designated calving area.	866	1.32	0.02	1.05–1.66
7—If 6%–10% of calvings in the last year occurred outside the designated calving area.	80	1.57	0.07	0.95–2.54
10—If greater than 10% of calvings in the last year occurred outside the designated calving area.	24	1.57	0.30	0.64–3.63
**How likely is it for calves to suckle multiple cows?**				
1—If no calves born on this farm ever suckle any cow.	937			
4—If 1%–10% of newborn calves suckle (i.e., happens quite rarely).	421	1.47	0.002	1.15–1.87
7—If 10%−50% of newborn calves suckle (e.g., only those calves born at night).	119	1.48	0.05	0.99–2.20
10—If more than 50% of newborn calves suckle (assume calves suckle if with the cow for more than 4 h or the owner deliberately leaves calves to suckle).	85	1.24	0.37	0.76–1.98
**Do you use someone else's equipment to spread slurry?**				
No	761			
Yes	808	1.22	0.06	0.99–1.52
**How often in the past year have neighbouring cattle broken into your cattle or your cattle broken into neighbouring cattle?**				
Never	960			
Once	376	1.33	0.03	1.03–1.72
Twice	188	1.61	0.004	1.16–2.23
3–5 times	40	2.26	0.009	1.20–4.24
More than 5 times	5	4.01	0.09	0.61–34.65
**Do you graze rented land?**				
No	463			
Yes	1106	1.92	<0.001	1.49–2.47
**Do your cattle come into contact with sheep?**				
No	954			
Yes	615	1.23	0.06	0.99–1.53
**Do/have you fed colostrum from other herds?**				
No	1560			
Yes	9	4.27	0.02	1.08–21.50

*Note*: *p*‐Value significant if *p* < 0.05. For risk factors scored 1, 4, 7 or 10, 1 was the reference score against which other risk scores were compared. For other risk factors ‘No’ and ‘Never’ was the reference response.

Logistical regression modelling retained six variables—‘Is the calving area used for lame or sick cows?’ (*p* > 0.001), ‘Do you graze rented land?’ (*p* > 0.001), ‘Are there single or multiple cows in the calving area?’ (*p* = 0.019), ‘Do JD‐positive or clinical cows calve in same area as the rest of the herd?’ (*p* > 0.001), ‘How often in the past year have neighbouring cattle broken into your cattle or vice versa?’ (*p* = 0.004) and ‘How many purchased animals were kept for breeding?’ (*p* = 0.001).

**FIGURE 3:**
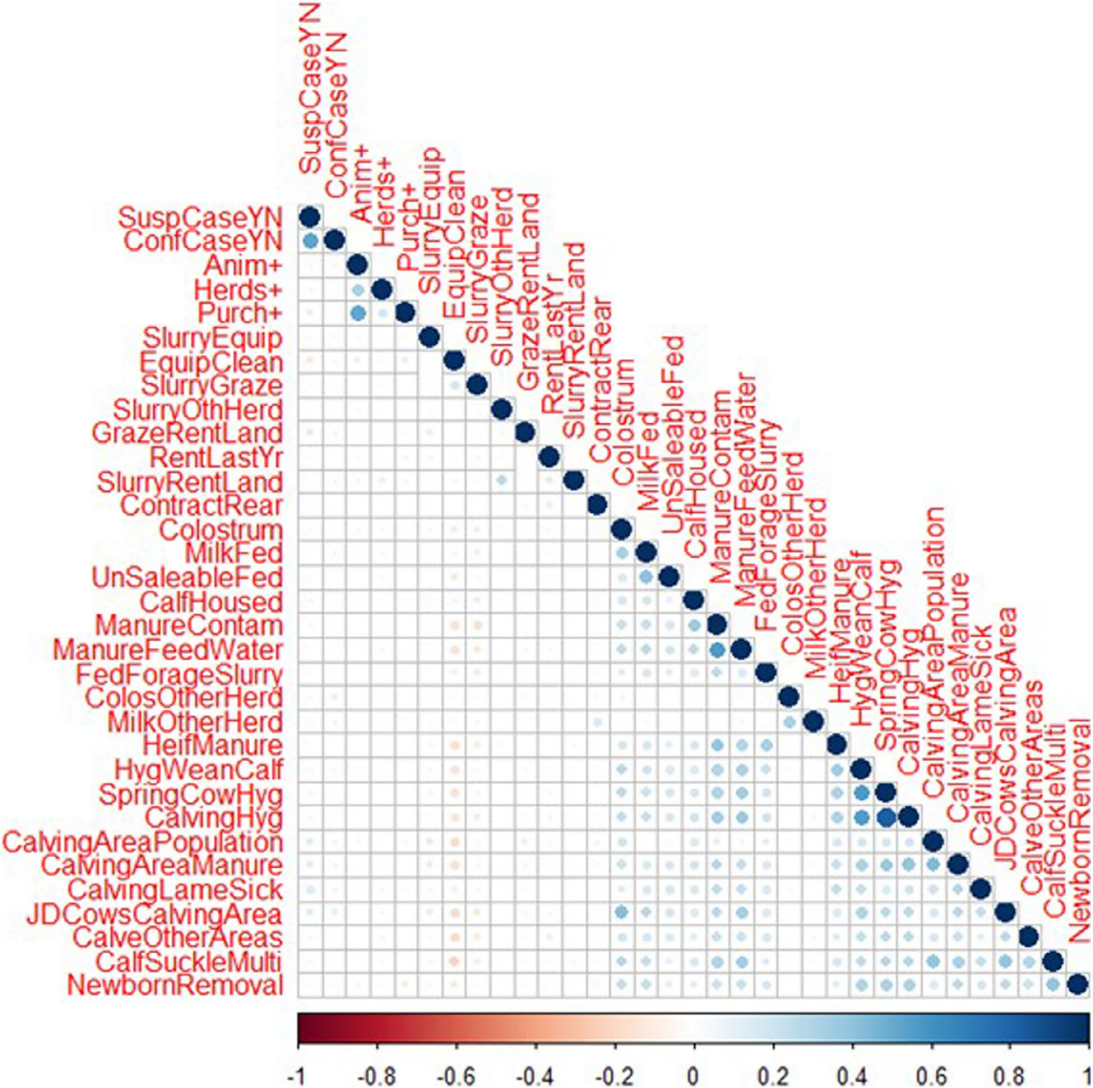
A correlation plot presenting the correlation (rho value) between risk factors (indicated by colour of circles) and correlation strength (indicated by area of circle) for all 1569 dairy herds in Northern Ireland that were analysed through the Veterinary Risk Assessment and Management Plan questionnaires.

Spearman's correlations reported rho values less than 0.3, suggesting that the retained variables were not highly correlated and were independent. Figure 3 shows the correlation plot for all VRAMP questions with a numerical answer that could be included in a correlation. A high correlation between high‐risk factor scores for multiple cow hygiene‐related risks was observed, with the highest positive correlation being shown between ‘How clean are the springing cows just before they enter the calving area?’ (SpringCowHyg) and ‘How clean are the cows at the point of calving down (i.e., after they enter the calving area)?’ (CalvingHyg). A strong positive correlation was observed between ‘Is there exposure to adult manure (cow and/or bull) in the calf housing or grazing area?’ (ManureContam) and ‘Is there exposure to cow manure by watering or feeding utensils?’ (ManureFeedWater).

## DISCUSSION

This study focused on the risk assessment stage of the NI JDCP, correlating farm management practices to transmission risks of JD. Identifying common risks will provide insight into areas that could be focused on as the control programme progresses and could develop into further research for dairy farms in NI. Risk factors were correlated with the presence of confirmed JD‐positive and suspected JD‐positive cows combined as ‘high probability of JD present’ herds due to the limited number of herds with a known JD status across the province.

Effective testing of individuals in the herd is an important component of JD control. Less than one‐quarter of herds in this study (21.4%) had undertaken any form of JD testing. Combining JD testing with milk recording, such as quarterly milk ELISA testing, can streamline the process for farmers, producers and laboratories, as well as reduce the possibility that JD‐positive cows are missed in the herd by providing frequent testing opportunities throughout the year.^17^ Within the NI herds, a higher percentage of herds reported having a suspected case of JD (29.8%) than the percentage of herds that had previously undertaken JD testing (21.4%), suggesting that approximately 8% of herds in the cohort did not check whether cows with suspected illness were infected with MAP by JD testing. These findings illustrate the need to undertake more widespread routine JD testing.

The adoption of biosecurity measures is important for both farm‐level and province‐wide JD control. Purchasing of cattle from infected herds or infected individuals is one of the main risk factors for JD transmission between herds.^18^ In Ireland, studies have shown that approximately 60%–80% of dairy herds purchase cattle to bring into the herd,^18^, ^19^ comparable to the data (89.4%) from this study, which shows that herds that report no animal introductions are a minority.^20^ Due to the lack of JD test information available at time of purchase, farmers are likely to purchase high or unknown JD risk animals and then manage risk of onward JD spread through quarterly milk testing. Multivariate logistical regression analysis of the VRAMP data found that farmers were significantly more likely to have identified a suspected case of JD within the herd if they had kept one or more purchased animals for breeding. Previous studies in Ireland in regard to on‐farm biosecurity measures, recommend that maintaining a closed herd is the most important factor to consider to provide optimum levels of disease prevention.^21^ If it is unsustainable to keep a fully closed herd, then ensuring that any animals brought into the herd are free from infection would be preferable to increase biosecurity.^22^ The current reported levels of testing (21.4% of herds) suggest that it will be difficult for many herds to assess, with confidence, their true infection status and therefore provide assurance of infection risk to purchasers of their livestock. However, other proxy measures of infection risk could be used, such as the number of animal introductions over time, which could be integrated into estimated herd infection assurance scores.^23^, ^24^

Other important recommendations for managing JD infection and preventing further infection in dairy herds include ensuring that JD‐positive, otherwise sick or lame cows are not housed or calved in calving pens with healthy, JD‐negative cows.^25^, ^26^ Approximately one‐quarter of participating NI dairy herds were at a high risk of JD in relation to both ‘Is the calving area used for lame or sick cows?’ and ‘Do JD‐positive or clinical cows calve in same area as the rest of the herd?’ (Figure 2). Previous studies in the USA found that 15%–30% of herds mix cows that are known to have a disease or have tested positive for MAP with healthy cows in the calving pen or utilise the calving pen as a hospital pen during the year.^27^, ^28^ Multivariate logistical regression analysis of our data set found that herds with a high‐risk score for either of the above risk factors were significantly more likely to be at a higher risk for having JD present within the herd as measured by the reporting of a confirmed or suspected case of JD. This suggests that across NI, the use of combined calving areas for sick and healthy animals is related to an increase in JD cases in herds. This should be taken into account as an area to focus on when discussing herd JD control plans.

Northern Ireland dairy herds that used grouped calving pens were significantly more likely to be at a higher risk of JD presence than herds that used individual calving pens (Table 3). Univariate analysis suggested that herds with a high level of manure contamination within the calving pen were three times more likely to be a ‘high probability of JD present’ herd (Table 3). Ensuring that the calving pens are thoroughly and frequently cleaned and re‐bedded is likely to reduce this risk of infection transmission at birth of a range of faecal/oral transmission pathogens.

The removal of calves from maternity pens as soon as possible after birth is recommended to reduce the exposure of calves to pathogens, including but not limited to MAP.^29^, ^30^ However, this measure was not widely adopted by herds within this study. Although 42% of herds were scored as high or very high risk of removing newborn calves from the dam at a slower rate than recommended for disease control, this risk factor was not found to significantly correlate with herds in the ‘high probability of JD present’ category. Although this should still be recommended as a control measure for JD, it appears that there are other risk factors that could be considered more important in relation to the NI JDCP, such as maternity and hospital pen management. There is also the potential that when herds suspect JD in the herd, they are more likely to follow the guidelines, which would reduce the correlation between the risk and cases.

Accidental mixing of herds through neighbouring cattle breaking into the farm or vice versa and herds that routinely grazed rented land were found to be significantly more likely to occur in herds that had a higher probability of JD present. Previous studies relating to bovine tuberculosis (bTB) have identified a link between herds that come into contact with infected neighbouring herds and a positive bTB test result.^31^, ^32^ In NI herds, previous studies have shown that cattle are four times more likely to have a positive bTB test if they have an infected neighbouring herd.^33^ However, it is unknown whether this is due to accidental mixing of herds, use of common land or sharing a common source of infection, such as a wildlife infection reservoir.^32^ While ensuring that neighbouring herds do not mix is a common biosecurity recommendation for JD,^34^ there are limited reports regarding the risk of mixing herds directly in relation to JD. The results of our study show that there is scope for further research into the reasons behind accidental mixing of neighbouring cattle being such a significant factor in JD transmission. For example, is the disease being spread directly through the mixing of animals, or are there underlying factors such as upkeep of farm boundaries that are also linked to the overall farm risk of JD transmission?

This study has several limitations. First, the JD infection status of any herd was not known at the time of VRAMP completion, so nothing can be said about timelines of infection. Data were only gathered on the most recent suspected case of JD in a herd, not positive cases, exact timings, or how many animals tested positive. Second, there were no data available on herd prevalence of JD at point of enrolment. Any identifying data, including herd size, previous ELISA results and numbers of cases, were not provided to ensure herd anonymity. Third, there was no information on whether herds had adopted JD relevant control measures prior to the VRAMP being conducted, because this is the first stage of the NI JDCP where any information has been documented. If any changes were made prior to the VRAMP completion, then these would have been based upon recommendations from a veterinarian or at the farmers discretion and they would not be included in any AHWNI captured data. Last, most importantly, a truly negative control group could not be defined. If this had been possible, then the significant risk factors may have been different.

This study has highlighted that calving pen management and hygiene, as well as herd biosecurity and land management, were all found to be significantly associated with MAP infection in NI dairy herds. The results of this study highlight the importance of general JD‐related management recommendations, such as separation of high‐risk animals and calving area management. These associations, alongside the general overview of JD in NI, support the importance of disease control programmes in dairy herds.

## AUTHOR CONTRIBUTIONS

Data analysed were collected by Animal Health and Welfare Northern Ireland, downloaded from the Wufoo platform and anonymised by Sam Strain before being provided to Kayleigh Meek. Kayleigh Meek reviewed, coded and collated data and carried out statistical analyses. Irene Grant, Niamh O'Connell and Sam Strain supervised Kayleigh Meek's work. All authors reviewed and edited the manuscript before submission.

## CONFLICTS OF INTEREST STATEMENT

Sam Strain is Chief Executive of Animal Health and Welfare Northern Ireland. The remaining authors declare they have no conflicts of interest.

## ETHICS STATEMENT

The authors confirm that the ethical policies of the journal, as noted on the journal's author guidelines page, have been adhered to. When herdowners registered with the Northern Ireland Johne's disease control programme, they signed an agreement giving consent that anonymised data about their herd could be shared with research organisations for the purposes of research relating to Johne's disease.

## Supporting information

S1. Details of Veterinary Risk Assessment and Management Plan (VRAMP) questions and codes used in statistical analyses of VRAMP responses.
S2. Johne's disease risk score guidance—provided to veterinarians trained by Animal Health and Welfare Northern Ireland to complete the Veterinary Risk Assessment and Management Plan (VRAMP) questionnaire.

## Data Availability

All of the research data are presented in the article.
