# Supplementary material for: Analysis of the Veterinary Risk Assessment and Management Plan questionnaire responses for dairy herds enrolled in the Northern Ireland Johne's disease control programme
Source: Vet Rec Open. 2023 Oct 9;10(2):e71. doi: 10.1002/vro2.71 (PMC10561023; doi:10.1002/vro2.71)
Supplement: Supplementary file 1 — S1. Details of Veterinary Risk Assessment and Management Plan (VRAMP) questions and codes used in statistical analyses of VRAMP responses. S2. Johne's disease risk score guidance—provided to veterinarians trained by Animal Health and Welfare Northern Ireland to complete the Veterinary Risk Assessment and Management Plan (VRAMP) questionnaire. [file VRO2-10-e71-s001.pdf]

Supporting information

S1 Details of Veterinary Risk Assessment and Management Plan (VRAMP) questions and codes used in statistical analyses of VRAMP responses.

Question	Analysis Code	Type of Question
**Have you ever completed a Johne's disease herd test?**	HerdTestYN	Yes or No (Closed Answer)
**If so, which test was used?**		
**Individual Milk ELISA**	IndMilk	Yes or No (Closed Answer)
**Individual Blood ELISA**	IndBlood	Yes or No (Closed Answer)
**Bulk Milk ELISA**	BulkMilk	Yes or No (Closed Answer)
**Faecal Culture**	FaeCul	Yes or No (Closed Answer)
**Faecal PCR**	FaePCR	Yes or No (Closed Answer)
**Have you had any suspect cases of clinical Johne's Disease e.g. cows with chronic diarrhoea/chronic wasting?**	SuspCaseYN	Yes or No (Closed Answer)
**How many suspect cases did you have?**	SuspCaseNum	Open Answer
**When was the most recent case?**	RecCaseYr	This Year, Last Year, 2 Years ago, 3 Years ago, More than 3 years ago (Closed Answer)
**Have you ever had a confirmed case (Faeces pos)**	ConfCaseYN	Yes or No (Closed Answer)
**Est. how many animals you have brought into the herd in last 5 years?**	AnimBrought	Open Answer
**Est. how many herds you have purchased from in last 5 years?**	HerdsBrought	Open Answer
**Est. how many purchased animals were kept for breeding?**	PurchBreeding	Open Answer
**Do you use someone elses equipment to spread slurry?**	SlurryEquipUseYN	Yes or No (Closed Answer)
**Is the equipment cleaned/flushed through?**	EquipCleanYN	Yes or No (Closed Answer)
**When using someone else's equipment/contractor - do you ensure that the first place spread with slurry is not pasture grazed by livestock less than 6 months of age?**	SlurryGrazeYN	Yes or No (Closed Answer)
**Do you spread slurry/manure from other herds on your pasture?**	SlurryOthHerdYN	Yes or No (Closed Answer)
**Do you graze cattle on commonage or with cattle from other herds?**	CommonageGraYN	Yes or No (Closed Answer)
**How often in the past year have neighbouring cattle broken into your cattle or your cattle broken into neighbouring cattle?**	NeighbCattYN	Never, Once, Twice, 3-5 Times, More than 5 times (Closed Answer)
**Do you graze rented land?**	GrazeRentLandYN	Yes or No (Closed Answer)
**Is there any land you use that you only started to rent in the last year?**	RentLastYrYN	Yes or No (Closed Answer)
**Is slurry/manure from other herds spread on rented land?**	SlurryRentLandYN	Yes or No (Closed Answer)
**Do you use contract rearers/B&B farms?**	ContractRearYN	Yes or No (Closed Answer)

**If so, do your cattle come into contact with/in the same airspace as other cattle?**	ContactOtherCattleYN	Yes or No (Closed Answer)
**Do your cattle come into contact with sheep?**	SheepYN	Yes or No (Closed Answer)
**Are cows fed colostrum from their own mother or from low risk cows, or artificial colostrum?**	ColostrumMotherorOther	1, 4, 7, 10 (Closed Answer)
**Are at least 3 litres of colostrum consumed within 2 hours?**	ThreeLitreColos	1, 4, 7, 10 (Closed Answer)
**Are heifer calves fed low risk whole, pasteurised or milk replacer?**	WholeorPastorReplace	1, 4, 7, 10 (Closed Answer)
**How often is un-saleable whole milk fed?**	UnSaleableFed	1, 4, 7, 10 (Closed Answer)
**Are calves housed in individual or group pens in the first week?**	IndorGroupFirstWeek	1, 4, 7, 10 (Closed Answer)
**Is there exposure to adult manure in calf housing or grazing areas?**	ManureHouseGraze	1, 4, 7, 10 (Closed Answer)
**Is there exposure to cow manure by watering or feeding utensils?**	ManureFeedWater	1, 4, 7, 10 (Closed Answer)
**Are calves fed forage which has received slurry from adult cows within last year?**	FedForageSlurry	1, 4, 7, 10 (Closed Answer)
**Do/Have you fed colostrum from other herds?**	ColosOtherHerdYN	Yes or No (Closed Answer)
**Do/Have you fed milk from other herds?**	MilkOtherHerdYN	Yes or No (Closed Answer)
**Are weaned heifers exposed to cows or manure?**	HeifManure	1, 4, 7, 10 (Closed Answer)
**What is the overall hygiene and cleanliness score of weaned cattle?**	HygWeanCalf	1, 4, 7, 10 (Closed Answer)
**How clean are the springing cows just before they enter calving area?**	SpringCowHyg	1, 4, 7, 10 (Closed Answer)
**How clean are the cows at the point of calving down?**	CalvingHyg	1, 4, 7, 10 (Closed Answer)
**Are there single or multiple cows in the calving area?**	CalvingAreaSingMult	1, 4, 7, 10 (Closed Answer)
**How much manure builds up in the calving area?**	CalvingAreaManure	1, 4, 7, 10 (Closed Answer)
**Is the calving area used for lame or sick cows?**	CalvingLameSick	1, 4, 7, 10 (Closed Answer)
**Do JD pos or clinical cows calve in same area as rest of herd?**	JDCowsCalvingArea	1, 4, 7, 10 (Closed Answer)
**What proportion of cows calve in areas other than calving pen?**	CalveOtherAreas	1, 4, 7, 10 (Closed Answer)
**How likely is it for calves to suckle multiple cows?**	CalfSuckleMulti	1, 4, 7, 10 (Closed Answer)
**How fast are newborn calves removed from their mothers?**	NewbornRemoval	1, 4, 7, 10 (Closed Answer)

S2 Johne's Disease Risk Score Guidance - provided to veterinarians trained by Animal Health and Welfare Northern Ireland to complete the Veterinary Risk Assessment and Management Plan (VRAMP) questionnaire

(Question 1 covered the herd's previous Johne's Disease (JD) testing status and did not require guidance.)

Q 2.1 Are calves fed colostrum from their own mother or from known low risk cows or artificial colostrum (artificial colostrum is recommended only in emergency situations)?

1 If all calves receive fresh clean colostrum only from their own test negative mother, or are fed colostrum from known low risk cows or artificial colostrum (this is only recommended in emergency situations), and if all calves born to a test positive mother are removed from the breeding programme permanently.

4 If all calves receive clean colostrum from only their own mother (no selection but no exposure of calves to cows other than their own mother) or are fed from a single low risk 'donor' cow (selected because test negative and older [≥8 yrs of age] and a record kept linking recipient calf to donor cow).

7 If colostrum from another cow(s) (pooled or frozen), with no selection based on JD status, is fed to 1 to 10% of calves.

10 If colostrum from another cow(s) (pooled or frozen) with no selection based on JD status is fed to more than 10% of calves.

Q 2.2 Are at least 3 litres of colostrum (first milking) consumed within the first 2 hours?

Feeding calves at least 3 litres of clean colostrum within the first two hours is recommended for effective passive immunity against common pathogens. While this is not directly linked to protection from JD, it is an essential element of calf rearing.

1 If every calf (bulls and heifers) gets at least 3 litres of clean colostrum (use 2 to 3 litres for Jersey calves) in the first 2 hours.

4 If more than 50% of all calves get at least 3 litres in the first 2 hours.

7 If less than 50% of all calves get at least 3 litres in the first 2 hours.

10 If less than 25% of all calves get at least 3 litres (due to insufficient volume being given).

Q 2.3 Are heifer calves fed on low risk whole milk, pasteurised low risk milk or milk replacer?

1 If calves have been fed milk replacer or pasteurised milk all the time for at least the past two years.

4 If calves are always fed whole milk selected from JD test negative or first calved heifers.

7 If calves are fed whole milk from individual cows but there is no selection e.g. based on JD test status.

10 If calves are fed whole milk from multiple cows e.g. from the bulk tank/rolling pools of milk.

Q 2.4 How often is non-saleable whole milk (high risk) fed?

1 If non-saleable milk is never fed.

4 If non-saleable milk is fed rarely e.g. once or twice per year.

7 If non-saleable milk is fed occasionally e.g. once or twice per month.

10 If non-saleable milk is fed routinely e.g. every week.

Q 2.5 Are calves housed in individual or group pens in the first week?

1 Calves are housed in single pens and away from the main cubicle / housing / calving area.

4 Calves are housed in single pens in the main cow cubicle or housing / calving area.

7 Calves are housed in groups ≤ 9.

10 Calves are housed in groups ≥10.

Q 2.6 Is there exposure to adult manure (cow and/or bull) in the calf housing or grazing area?

1 Calf housing, pasture and feeding is entirely separate from any manure (or slurry) from adult animals or adult cattle transit areas (including using different equipment and wearing different clothing and boots).

4 Calves are housed or at pasture near adult animals but there is no possibility of contamination by manure (or slurry) from adult animals being carried by adult animals or people (i.e. boots are routinely washed when moving from cows to calves, or calves are looked after before any cow handling occurs, etc.), i.e. while calves are close to adult animals there is some deliberate attempt to maintain a 'barrier.'

7 Calves are housed or at pasture near cows (or followers) and are exposed via cow traffic areas (roadways) or via people movement (i.e. there is no attempt to maintain a barrier).

10 Calves are housed or grazed near cows (or where slurry was spread), share feeding areas or water: direct contamination or contact is likely (i.e. there is no barrier and contact risk is high).

Q 2.7 Is there exposure to cow manure by watering or feeding utensils?

1 If mixing utensils and feed/water buckets are visibly clean and all are washed daily with detergent or disinfectant or used exclusively for milk replacer feeding and have no exposure to manure from adult animals.

4 If trace amounts of manure are visible or mixing utensils/buckets are washed less frequently than daily but at least weekly.

7 Regardless of cleaning practices, manure is clearly visible.

10 Regardless of cleaning practices, manure contamination is extensive.

Q 2.8 Are calves fed forages that have received slurry from adult animals within the last year?

1 Grass/silage/other forages fed to calves not spread with slurry/ farm yard manure from adult animals in the previous 12 months.

4 Hay fed to calves spread with slurry/ farm yard manure from adult animals in the previous 12 months.

7 Silage/other conserved forages fed to calves spread with slurry/ farm yard manure from adult animals in the previous 12 months.

10 Grass fed to calves spread with slurry/ farm yard manure from adult animals in the previous 12 months.

Q 3.1 Are weaned heifers exposed to cows or their manure at any time?

1 If never housed or grazed with or near manure from adult animals, have no direct contact, or exposed to manure from adult animals by run-off or slurry spreading, are not fed uneaten rations from cows and not sharing cow water troughs.

4 If housed or grazed near cows for only a short time, no direct contact, no exposure to cow manure by run-off or slurry spreading, not fed uneaten rations from cows and are not sharing cow water troughs.

7 If housed or grazed near cows, direct contact possible or exposed to cow manure by run-off or slurry spreading, or fed uneaten rations from cows, or sharing cow water troughs.

Q 3.2 What is the overall heifer hygiene and cleanliness score?

1 If heifers have no manure visible on hindlegs, forelegs or flanks.

4 If manure is present on hind or forelegs but not above hock/carpal joints.

7 If manure is present on hind or forelegs above hock/carpal joints and/or is present on the flanks.

Q 4.1 How clean are the springing cows just before they enter the calving area?

1 If no cows have manure visible on hindlegs, forelegs or flanks.

4 If manure is present on hind or forelegs but not above hock/carpal joints.

7 If manure is present on hind or forelegs above hock/carpal joints and is present on the udder or flanks of a few cows (<10%).

10 If manure is present on hind or forelegs above hock/carpal joints and is present on the udder or flanks of a significant proportion of cows (>10%).

Q 4.2 How clean are the cows at the point of calving down (i.e. after they enter the calving area)?

1 If no cows have manure visible on hindlegs, forelegs or flanks.

4 If manure is present on hind or forelegs but not above hock/carpal joints.

7 If manure is present on hind or forelegs above hock/carpal joints and is present on the udder or flanks of a few cows (<10%).

10 If manure is present on hind or forelegs above hock/carpal joints and is present on the udder or flanks of a significant proportion of cows (>10%).

Q 4.3 Are there single or multiple cows in the calving area?

1 If there is never more than one cow in the calving pen/area.

4 If <25% of the time, there is more than one cow in the calving pen/area.

7 If 25% – 50% of the time, there is more than one cow in the calving pen/area.

10 If more than 50% of the time, there is more than one cow in the calving pen/area.

Q 4.4 How much manure builds up in the calving area and therefore what risk is there of calf exposure to faecal pathogens?

1 Pen is cleaned out between each calving.

4 No visible manure, pen has not been cleaned between every calving, but new bedding is added so that the bedding is dry.

7 Visible manure covering some of the floor (less than 50%).

10 Visible manure covering most of the floor (more than 50%).

Q 4.5 Is the calving area used for lame or sick cows?

1 If the area is NEVER used by non-calving cows.

4 If the area is used rarely (e.g. once a quarter).

7 If the area is used occasionally (e.g. once a month).

10 If the area is used routinely (e.g. it is the usual place where sick/lame/treated animals are held).

Q 4.6 Do Johne's Disease clinical or test positive cows calve in the same area as the rest of the herd?

1 No JD clinical, test positive, suspect or high-risk cows have contact with calving pens used by other cows. Such high-risk cows are calved in a separate location.

4 Rarely a test positive, suspect or high-risk cow calves in the general calving area but never any animal showing clinical signs.

7 Frequently test positive, suspect or high-risk cow calves in the general calving area but never any animal showing clinical signs.

10 There is no segregation at calving between high risk JD clinical, test positive, suspect or high risk and low risk cows.

Q 4.7 What proportion of the cows calf in areas other than the designated area (e.g. in cubicle houses)?

1 If no calves are born anywhere other than in the designated calving area/pen/on clean grass in the last year.

4 If 0 to 5% of calvings in the last year occurred outside the designated calving area.

7 If 6% to 10% of calvings in the last year occurred outside the designated calving area.

10 If greater than 10% of calvings in the last year occurred outside the designated calving area.

Q 4.8 How likely is it for calves to suckle cows?

1 If no calves born on this farm ever suckle any cow.

4 If 1-10% of newborn calves suckle (i.e. happens quite rarely).

7 If 10 -50% of newborn calves suckle (e.g. only those calves born at night).

10 If more than 50% of newborn calves suckle (assume calves suckle if with the cow for more than 4 hours or the owner deliberately leaves calves to suckle).

Q 4.9 How fast are newborn calves removed from their mothers?

1 If >90% of newborn calves are removed within 15 minutes of birth.

4 If >50% of newborn calves are removed within 30 minutes (likely to only miss those born at night).

7 If 10 – 50% of newborn calves are removed within 30 minutes.

10 If less than 10% of newborn calves are removed within 30 minutes.
